# Transabdominal Migration of Retained Surgical Sponge

**DOI:** 10.1155/2012/249859

**Published:** 2012-10-16

**Authors:** Ali Guner, Gultekin Hos, Izzettin Kahraman, Can Kece

**Affiliations:** Department of General Surgery, Trabzon Numune Training and Research Hospital, 61040 Trabzon, Turkey

## Abstract

Retained surgical sponge (RSS) is a rare surgical complication. The RSSs are mostly located intra-abdominally but they can also be left in the thorax, spine, extremity, cranium, and breast. RSS is often difficult to diagnose because of the nonspecific clinical symptoms and radiologic findings. Clinically, RSS may present as an exudative reaction in the early postoperative period or may also cause an aseptic fibrous tissue response. A foreign body may remain asymptomatically silent for a long time, and it may later present with obstruction, fistulization, or mass formation. In this report, we present a case in which an RSS has migrated through the abdominal wall and caused an anterior abdominal wall abscess.

## 1. Introduction

Retained surgical sponge (RSS) is a rare surgical complication. A foreign body may remain asymptomatically silent for a long time, and it may later present with obstruction, fistulization, or mass formation. In this report, we aimed to present a case in which an RSS has migrated through the abdominal wall and caused an anterior abdominal wall abscess and clinically sepsis.

## 2. Case Presentation

**Figure 1:**
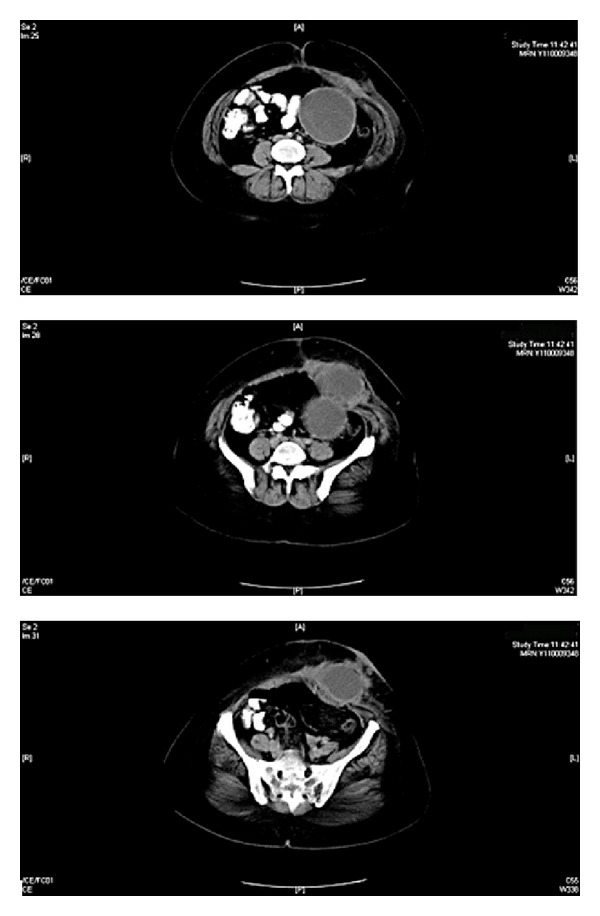
Three different views of computerized tomography show a mass formation extending to the abdominal wall.

**Figure 2:**
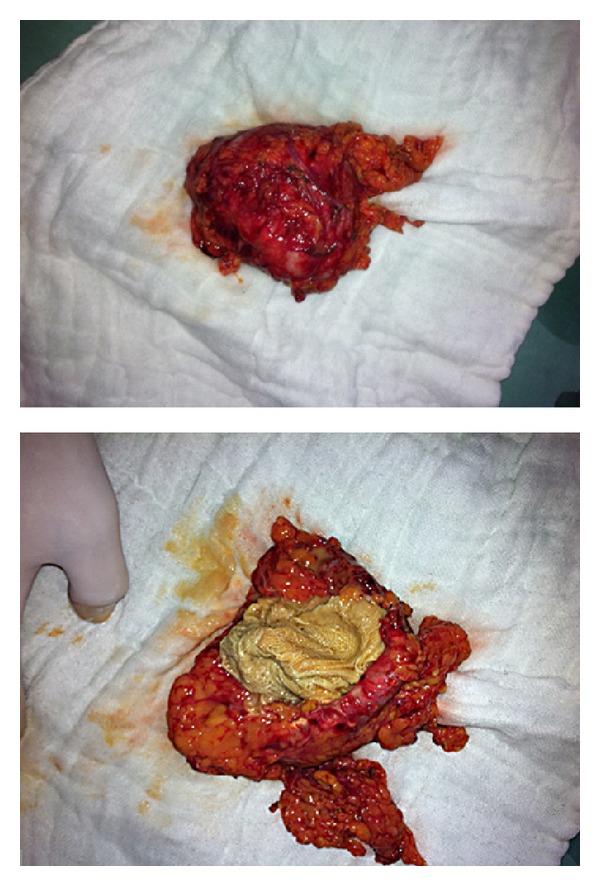
A sponge surrounded by a granulation tissue.

A 27-year-old woman was admitted to our surgical unit with a series of symptoms including left-sided abdominal colic lasting for a month and stiffness, increasing erythema, and purulent discharge which had developed in the past week. The patient was septic and her body temperature was 38.1 degrees centigrade. In the abdominal examination, a firm, mobile, and tender mass with purulent discharge was palpable on the left side of the umbilicus. Blood analysis showed a marked leukocytosis (WBC: 17000) and increased CRP: 15.4 (normal range: 0–0.5). Contrast-enhanced abdominal CT revealed a mass formation located intra-abdominally, neighboring the small intestine, and extending to the skin through the abdominal wall (Figure 1). The patient had no evidence of any obstruction. She had undergone a cesarean section operation two years ago, and there was not any other significant event in her history. RSS was suspected and a laparotomy was planned. At the laparotomy, there was a mass formation surrounded with granulation tissue penetrating the abdominal rectus muscle extending to the subcutaneous level and causing a cutaneous fistula. Purulent collection was present around the mass. The mass had no relation with either the intestinal segments or other intra-abdominal organs. The mass was excised including the skin and the subcutaneous tissues. Prosthetic material was not applied because of the infection, and the incision was closed primarily. The excised mass was evaluated, and a sponge surrounded by a granulation tissue was observed (Figure 2). Histopathological assessment was reported as granulation due to the foreign body. The postoperative period was uneventful, and the patient was discharged on the fifth day of her hospitalization.

## 3. Discussion

RSS is also known as gossypiboma, meaning a mass of cotton matrix retained within the human body (from the Latin word “Gossypium,” meaning “cotton” and the Swahili word “boma,” meaning “place of concealment”) [1]. It is also a derivative of the word “gossip” (because of causing gossip about the surgeon). The first case was described by Wilson in 1884, and since then this avoidable mistake has continued to be a serious problem among surgeons in spite of the developments in technology and surgery. RSS cases have been reported for almost all organs. This event is mostly observed in general surgery operations. Gynecological (22%), urological (10%), and orthopedic surgeries (6%) are the other departments where the same situation may also occur. The abdominal cavity is the most involved region and the thorax is the second one. Surgical instruments, needles, clamps, and retractors are all potentially forgettable objects but surgical sponges are the most retainable ones [2]. In this case, we present a patient with a retained sponge after a gynecological operation, and we evaluated the risks and the complications of this event.

RSS results in a broad spectrum of clinical presentations depending on the location of the sponge and the type of reaction. The reported time to diagnosis ranges from 1 day to 40 years. They are inert materials and do not cause any specific biochemical reactions other than granulation reaction so that they can stay asymptomatic for a long time. They cause two different tissue reactions that shape the clinical presentation. The first one is the exudative pattern, usually present in the early postoperative period surgical site infection is the most important clinical finding in this period and can be complicated with wound infection, abscess, fistula formation, or intra-abdominal sepsis, causing high mortality. The second type of reaction is the fibrinous response. This type of reaction, observed in 25% of cases, is formed as a result of encapsulation of the retained foreign body by the scar tissue and granuloma formation. This aseptic reaction may mimic a pseudotumor form or cause intestinal obstruction and erosion of urinary or gastrointestinal tissues [3]. Foreign bodies have been reported which have migrated to the gastrointestinal organs and have been thrown out by feces. Various hypotheses have been proposed to explain how the presumed transvisceral migration of such a foreign body might occur. Dhillon and Park suggested that an inflammatory reaction surrounds the foreign body, and an abscess pouch forms and erodes the neighboring tissues [4]. Following this process, it is suggested that the foreign body moves forward by the peristalsis. This migration may occur mostly transileally but cutaneous fistulization through the anterior abdominal structures is also another alternative route. In this case, we present an RSS which has migrated transabdominally and caused an exudative reaction in the two-year postoperative period.

The diagnosis of a retained surgical foreign body is based upon imaging studies which demonstrate the object. Plain radiographs are the first used imaging methods but their accuracy is not very high. They are not favorable because of the impaired film quality, different radiopaque image, and a lack of awareness or fragmentation of the radio-opaque markers. Thus, computed tomography is preferred as the initial study. A low-density heterogeneous mass with a spongiform pattern that contains gas bubbles is the characteristic appearance of RSS on computed tomography. If necessary, gastrointestinal contrast studies, ultrasonography (for extremity surgery), magnetic resonance imaging (for spinal surgery), mammography (for breast surgery), or positron emission tomography are the other important radiologic modalities which can be helpful [5, 6].

Removal of RSS is indicated to prevent potential complications. Especially long existing asymptomatic RSS cases can be followed after being informed about the potential complications but surgical removal of the sponge should be recommended. Although RSS can be excised laparoscopically, laparotomy is usually preferred due to the size of the foreign body and the adhesions surrounding it [7]. Transmurally migrated cases can be treated endoscopically or extraction of the foreign body spontaneously via the rectum can be a cure. In our case, the foreign body had migrated to subcutaneous tissues transabdominally and was excised with the nearby structures by open surgery.

The recognition of risk factors for this undesirable complication would be of particular importance for the surgeons. Emergency surgical operation is the most important risk factor that increases the RSS risk 9 times. However, RSS often occurs after elective surgery (70%). High body mass index and an unplanned change in the type of surgery are the other important risk factors. Although prevention strategies such as standardized count protocols, radiographic screening in the operating room, counting devices such as bar codes or radiofrequency chips are used in several centers, this complication still exists [8].

In conclusion, RSS must be remembered in the differential diagnosis of the patients presenting with intra-abdominal mass and declaring a past operation. Clinical suspicion and suitable imaging methods constitute the most important steps for the diagnosis. RSS cases may be asymptomatic for a long time but can also cause lethal complications which can be accompanied by serious medicolegal problems.
